# Sintilimab plus nab-paclitaxel as second-line treatment for advanced biliary tract cancer: study protocol for an investigator-initiated phase 2 trial (NapaSinti trial)

**DOI:** 10.1186/s12885-023-11188-4

**Published:** 2023-08-07

**Authors:** Nan Zhou, Xiaofen Li, Yu Yang, Sirui Tan, Shunyu Zhang, Qiyue Huang, Hongfeng Gou

**Affiliations:** 1 Department of Medical Oncology, Cancer Center, West China Hospital, Sichuan University, No.37 Guo Xue Xiang, 610041 Chengdu, Sichuan China; 2 Division of Abdominal Tumor Multimodality Treatment, Cancer Center, West China Hospital, Sichuan University, Chengdu, China; 3 Gastric Cancer Center, West China Hospital, Sichuan University, Chengdu, China

**Keywords:** Biliary tract cancer, Immunotherapy, anti-PD-1 antibody, Sintilimab, Chemotherapy, Nanoparticle albumin-bound (nab)-paclitaxel, Second line, Phase 2 study

## Abstract

**Background:**

Biliary tract cancer (BTC) is a relatively rare but highly aggressive malignancy. However, there is currently no satisfactory second-line regimen for patients without specific genetic mutations. Nanoparticle albumin–bound paclitaxel, also known as nab-paclitaxel (Abraxane, Bristol Myers Squibb), has shown activity in patients with BTC. Studies investigating the immunogenic features of BTC suggested that checkpoint inhibition may lead to antitumor immune responses. In recent years, improved survival has been observed in patients treated with chemotherapy combined with immunotherapy across multiple cancer types, including BTC. This clinical trial aims to evaluate the efficacy and safety of second-line sintilimab in combination with nab-paclitaxel in advanced BTC patients.

**Methods:**

The NapaSinti trial is a prospective, nonrandomized, open-label, phase 2 study conducted at a tertiary hospital in Chengdu, China. Eligible patients are those with histologically or cytologically confirmed locally advanced non-resectable or metastatic adenocarcinoma in the biliary tract (including intrahepatic cholangiocarcinoma, extrahepatic cholangiocarcinoma, and gallbladder cancer), aged between 18 and 75 years, with an Eastern Cooperative Oncology Group (ECOG) performance status of 0 or 1, who have experienced disease progression after prior gemcitabine- or fluorouracil-based chemotherapy and have not received taxane or immune checkpoint inhibitor treatment. Enrolled patients will receive intravenous administration of sintilimab 200 mg on day 1 and nab-paclitaxel 125 mg/m2 on days 1 and 8, every three weeks. The primary endpoint is the objective response rate (ORR), while the secondary endpoints include overall survival (OS), progression-free survival (PFS), and safety. Exploratory objectives aim to identify biomarkers and molecular signatures for predicting response or prognosis. Using Simon’s two-stage design, a total of 63 participants will be enrolled in the study. This trial was initiated in March 2022 in China.

**Discussion:**

The NapaSinti trial evaluates the efficacy and safety of second-line sintilimab plus nab-paclitaxel for advanced biliary tract cancer. Additionally, the trial provides an opportunity for translational research.

**Trial registration:**

Chinese Clinical Trial Registry ChiCTR2100052118. Registered October 19, 2021.

## Background

Patients with advanced biliary tract cancer (BTC) have limited options for second-line treatment [1]. The ABC-06 study, the only phase 3 trial conducted in this setting, demonstrated that the median overall survival (mOS) of active symptom control (ASC) plus FOLFOX (folinic acid, fluorouracil, and oxaliplatin) group is merely 0.9 months longer than that of the ASC group (6.2months vs. 5.3months; P = .031) [2].

Previous studies have suggested the clinical benefit of nanoparticle albumin-bound (nab)-paclitaxel in BTC [3, 4]. In a single-arm, multicenter trial, combination chemotherapy with capecitabine and nab-paclitaxel demonstrated feasibility as a regimen for advanced BTC in patients who had progressed on first-line chemotherapy, with a disease control rate of 80% and overall survival of 12.1 months [5]. Recently, early-phase data from the ongoing phase 3 SWOG S1815 trial demonstrated improvements in median PFS and OS when nanoparticle albumin–bound (nab) paclitaxel is combined with gemcitabine-cisplatin (GC) in the first-line setting [6].

Sintilimab, an anti-PD-1 antibody, has exhibited high affinity to human PD-1 and superior PD-1 occupancy in circulating T cells [7]. Pembrolizumab or nivolumab monotherapy was well tolerated and provided modest efficacy with durable response in patients with BTC [8, 9].

It is well-accepted that chemotherapy enhances antitumor activity through direct or indirect immune-system stimulation [10]. Martin JD, et al. further demonstrated the potential of nanomedicines to enable durable responses to immunotherapy [11]. Improved outcomes and manageable safety profiles have been observed with immune checkpoint inhibitors (ICIs) combined with nab-paclitaxel in breast cancer, pancreatic adenocarcinoma, urothelial carcinoma, and other malignancies [12–14]. Currently, durvalumab is approved in combination with gemcitabine and cisplatin for the first-line treatment of advanced BTC. Based on these findings, investigating the efficacy and safety of sintilimab in combination with nab-paclitaxel as a second-line treatment for advanced BTC is warranted.

The NapaSinti trial aims to challenge the standard second-line therapy for advanced BTC by evaluating the combination of sintilimab and nab-paclitaxel, and it also provides an opportunity for translational research.

## Methods and design

This is a prospective, single-arm, open-label, phase 2 study designed to evaluate the efficacy and safety of second-line treatment with sintilimab in combination with nab-paclitaxel in patients with advanced biliary tract cancer (BTC).

### Patient selection

The main inclusion criteria for this study are as follows: histologically or cytologically confirmed advanced adenocarcinoma in the biliary tract; previous treatment with gemcitabine or fluorouracil-based chemotherapy as first-line therapy; not received taxane or immunotherapy; age between 18 and 75 years; life expectancy of at least 3 months; Eastern Cooperative Oncology Group (ECOG) performance status of 0 or 1; measurable lesions according to the Response Evaluation Criteria in Solid Tumors guidelines version 1.1 (RECIST 1.1); and adequate organ function. Complete inclusion and exclusion criteria are detailed in Table 1.

**Table 1:** Eligibility criteria

Key Inclusion Criteria	Key Exclusion Criteria
1. Histologically or cytologically confirmed locally advanced non-resectable or metastatic adenocarcinoma in the biliary tract, including intrahepatic cholangiocarcinoma, extrahepatic cholangiocarcinoma, and gallbladder cancer; 2. Aged 18 to 75 years, regardless of gender; 3. Expected life expectancy of ≥ 3 months; 4. ECOG PS of 0 or 1; 5. Documented disease progression after previous gemcitabine or fluorouracil-based systemic chemotherapy; not received any taxane therapy, including but not limited to paclitaxel, paclitaxel liposome, nab-paclitaxel, and docetaxel; 6. Measurable disease according to RECIST version 1.1. Assessment should be carried out within 28 days prior to study enrollment; 7. Adequate hematologic, hepatic, and renal functions (within 7 days before study entry): a. Hemoglobin ≥ 90 g/L b. Neutrophils ≥ 1500/mm^3^ c. Platelets ≥ 75,000/mm^3^ d. Aspartate aminotransferase and alanine aminotransferase ≤ 3.0 x ULN, or ≤ 5.0 x ULN in case of liver metastasis e. Bilirubin ≤ 1.5 x ULN f. Creatinine ≤ 1.5 x ULN g. Activated partial thromboplastin time, prothrombin time, and international normalized ratio ≤ 1.5 x ULN h. No prior blood transfusion, granulocyte colony-stimulating factor, or other medical support therapy; 8. In case of active hepatitis B or C, antiviral therapy starting at least 14 days before experimental drug administration and hepatitis B virus DNA ≤ 2500 copies/mL or ≤ 500IU/mL and hepatitis C virus RNA within the lower limit of detection; 9. Voluntarily participate and sign an informed consent.	1. Histologically confirmed squamous-cell carcinoma, adenosquamous carcinoma, undifferentiated carcinoma, or sarcoma; 2. Malignancies of duodenal ampulla; 3. Severe hepatic or renal insufficiency; myocardial infarction within 3 months; 4. Patients with severe uncontrolled medical conditions or acute infections (infection-induced fever above 38 °C); 5. History of another malignancy with disease-free survival of less than 5 years, except for curative in situ cervical cancer, curative skin basal cell carcinoma, and curative gastrointestinal cancer by endoscopic mucoresection; 6. Current or past history of autoimmune diseases, including but not limited to interstitial lung disease, uveitis, enteritis, nephritis, hyperthyroidism, and hypothyroidism; 7. Active pulmonary tuberculosis within one year; 8. Suffering from interstitial lung disease or non-infectious pneumonia, or previous lung diseases may hinder the assessment or management of pulmonary toxicity associated with study drugs; 9. Severe infection requiring intravenous antibiotics, antifungal agents, or antiviral drugs; 10.Patients with a long history of chronic diarrhea or those with complete intestinal obstruction present; 11. Pregnant or breastfeeding female; unwillingness to use contraceptive measures in males and females; 12. Immunosuppressant or corticosteroid (systemic or local) used to suppress immune function within 2 weeks before inclusion; except for local or physiological doses of systemic glucocorticoids (e.g., no more than 10 mg/day of prednisone or other glucocorticoids of equivalent dose) by nasal spray, inhalation or other routes, or hormones used to prevent allergy; 13. Previous treatment with immunotherapy, such as anti-PD-1, anti-PD-L1, anti-PD-L2, or any other T-cell co-stimulation or checkpoint inhibitor therapy; 14. History of allergic disease or severe hypersensitivity to the experimental drugs; 15. Congenital or acquired immunodeficiency, such as HIV infection; 16. Other conditions deemed unsuitable for participation in this study by the investigators.

Abbreviations: ECOG, Eastern Cooperative Oncology Group; PS, performance status RECIST, Response Evaluation Criteria in Solid Tumors; ULN, upper limit of normal; PD-1, programmed cell death-1; PD-L1, programmed cell death-ligand 1; HIV, human immunodeficiency virus

### Treatment

1. Sintilimab: 200 mg, administered as a 30–60 min intravenous infusion on day 1, every three weeks.
2. Nab-paclitaxel: 125 mg/m^2^, initiated ≥ 30 min after sintilimab, administered as a 30–40 min intravenous infusion on day 1 and day 8, every three weeks.

**Fig. 1:**
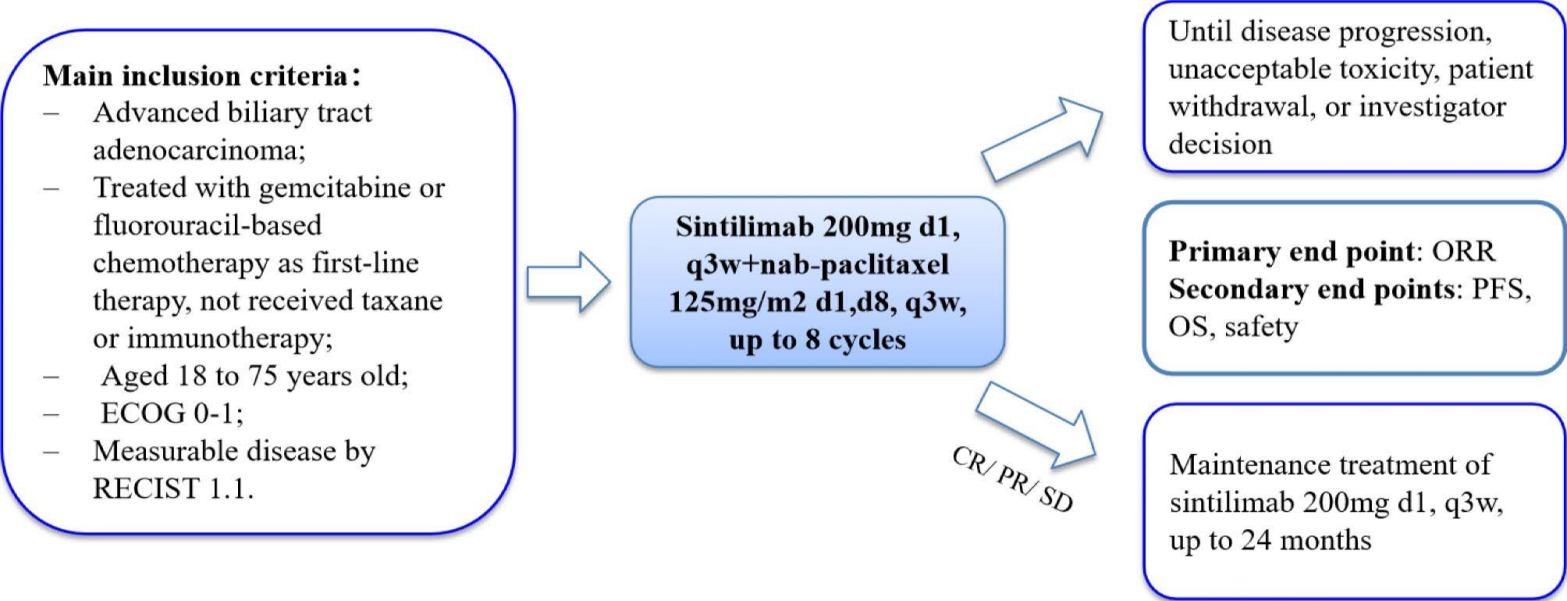
Outline of the study design

Eligible patients will receive a total of eight cycles of sintilimab plus nab-paclitaxel. Patients who do not experience disease progression or unacceptable toxicity will continue with sintilimab monotherapy maintenance for up to 24 months. Treatment must adhere to all protocol-related requirements. The planned enrollment period for the study is two years, from March 30, 2022, to March 30, 2024, and the planned follow-up period is two years from the date of enrollment of the last patient. The study design is illustrated in Fig. 1.

### Treatment discontinuation

Treatment will be discontinued for any of the following reasons:.

1. The patient declines further study treatment or withdraws consent to participate.
2. Progressive disease is confirmed by the investigator.
3. Unacceptable toxicity is determined by the patient or investigator.
4. Exclusion criteria are met.
5. The patient fails to comply with the protocol.
6. The investigator determines that the continuation of treatment is not in the best interest of the patient.

The reasons for discontinuing treatment will be documented in the patient’s medical records.

### Study endpoints

#### Primary endpoint

Objective response rate (ORR), defined as the proportion of complete responses (CRs) and partial responses (PRs).

#### Secondary endpoints

1. Overall survival (OS), defined as the time from starting second-line therapy until death or last follow-up.
2. Progression-free survival (PFS), defined as the time from starting second-line therapy until progression or last follow-up or death from any cause.
3. Safety assessment according to the Common Terminology Criteria for Adverse Events (CTCAE) version 5.0.

#### Assessments

Complete blood cell count (red blood cell, hemoglobin, platelet count, white blood cell), measurement of liver and renal function, and electrolyte panel are performed weekly. The following assessments are performed before each cycle: a complete blood cell count, measurement of liver and renal function, electrolyte panel, coagulation parameters, myocardial enzymogram, thyroid-related hormones, 12-lead electrocardiogram. Morphological imaging (contrast-enhanced computed tomography (CT) of the abdomen and chest) and biological tumor assessments (carcinoembryonic antigen (CEA), carbohydrate antigen 19 − 9 (CA199), carbohydrate antigen 125 (CA125)) are conducted every six weeks. The response is determined using CT scanning based on RECIST 1.1.

Health-related quality of life (HRQoL) questionnaires will be completed before each cycle. HRQoL questionnaires are based on assessments using the European Organization for Research and Treatment of Cancer Quality of Life Questionnaire Core 30 (EORTC QLQ-C30) and the Cholangiocarcinoma and Gallbladder Cancer module (QLQ-BIL21)..

The investigators will monitor adverse events in every circle. All adverse events observed during the study treatment period are appropriately registered in the subjects’ medical records and electronic case report forms. Adverse events will be assessed according to the CTCAE version 5.0.

### Follow-up

After treatment, regular follow-up is scheduled every three months. If necessary, follow-up may be conducted more frequently through telephone interviews or face-to-face visits. The follow-up period continues until death or the end of the study, regardless of the duration of treatment. The following procedures are performed during follow-up:.

1. Assessment of survival status.
2. Completion of health-related quality of life (HRQoL) questionnaires.
3. Recording of all adverse events.

### Biomarker analysis

Tumor tissue and blood samples are collected before treatment. The serum is analyzed for cytokines, while the tumor tissue is used to explore immune cell populations and the expression of selected tumor markers. Tumor samples will also be analyzed with next-generation sequencing (NGS), which allows comprehensive genomic profiling of critical targeted therapy and immunotherapy biomarkers, such as tumor mutation load, programmed cell death ligand 1 (PD-L1) expression, and microsatellite status. This analysis aims to identify biomarkers and molecular signatures that can predict treatment response or prognosis.

### Sample size calculation and statistical analysis

The sample size calculation was performed using Simon’s optimal two-stage design [15] with an α error of 5% and a power of 80%. ABC-06 clinical trial showed that the ORR for second-line treatment of advanced biliary tract cancer was 5% [2]. The expected number of patients was calculated according to the alternative hypothesis that the objective response rate would be 15% or higher and the null hypothesis that the ORR would be 5% or lower. The trial consists of two stages: stage 1 requires the enrolment of at least 23 patients, with at least 1 patient achieving PR or CR. Once this criterion is met, stage 2 will be initiated, with the enrolment of at least 33 additional participants to achieve a sample size of 56 patients. Considering a 10% loss to follow-up rate, the actual study requires a total sample size of 63 subjects. Overall, if at least five patients had a response, the treatment regimen would be deemed a success. The analysis will be performed on an intention-to-treat basis, where all patients will be included in the analysis, and missing data will be considered as treatment failure. The sample size calculation was performed using the “Two-stage designs for tests of one proportion (Simon)” in PASS 2021 software (version 21.0.3 NCSS, LLC. Kaysville, Utah, USA, ncss.com/software/pass).

### Confidentiality

All study-related information will be securely stored at the study site. Participants’ information will be kept in locked file cabinets located in restricted-access areas. To maintain participant confidentiality, all laboratory specimens, reports, data collection, process, and administrative forms will be identified only by a coded identification number.

## Discussion

Patients with advanced biliary tract cancer (BTC) have a poor prognosis, with a median OS of approximately 12 months [16]. A systematic review of second-line chemotherapy for advanced BTC that included 761 patients reported an OS of 7.2 months [95% confidence interval (CI) 6.2–8.2], a PFS of 3.2 months (95% CI 2.7–3.7), and response rate of 7.7% (95% CI 4.6–10.9) [17]. Although patients with molecular alterations such as HER2, BRAF, FGFR2, and IDH1 may benefit from targeted therapies, drugs are expensive and poorly accessible in China. Additionally, only a small number of patients have these specific genetic mutations [18–21]. A study has revealed the presence of CD8 + tumor-infiltrating lymphocytes and PD-L1 expression on cancer cells in BTC [22], providing support for the potential use of ICIs in BTC. Nevertheless, patients with advanced BTC failing standard treatment have shown limited benefits from pembrolizumab monotherapy, with durable antitumor activity observed in only 6–13% of patients [8]. Although combination immunotherapy with PD-1/PD-L1 and CTLA-4 blockade has shown some advantages over single-agent immunotherapy, it has not resulted in substantial improvements in overall survival [23]. Furthermore, the combination of immunotherapy and targeted therapy has demonstrated minimal benefits in BTC [24–26]. Therefore, the development of novel therapeutic combinations that can extend the clinical benefits for advanced BTC remains a significant challenge.

Sintilimab, a highly selective, fully humanized, anti-PD-1 antibody, blocks the interaction between PD-1 and its ligands. The ORIENT-12 and ORIENT-32 studies indicated that the addition of sintilimab offers clinical benefits for non-small cell lung cancer and unresectable hepatocellular carcinoma, with an acceptable safety profile [27, 28]. It has been suggested that chemotherapy and immunotherapy have a synergistic effect by overcoming the immunosuppressive effects of the tumor microenvironment, increasing the cross-presentation of tumor antigens, and facilitating better penetration of immune cells into the tumor core [29]. Another study revealed that the immune response stimulated by durvalumab could be further enhanced by chemotherapy-induced immunogenic cell death [30]. Of note, chemoimmunotherapy has already shown encouraging results in advanced BTC. A phase 1 study demonstrated that nivolumab plus gemcitabine-cisplatin (GC) in patients with unresectable or recurrent biliary tract cancer were associated with signs of antitumor activity [31]. Additionally, the TOPAZ-1 phase 3 study indicated that durvalumab plus GC as first-line treatment significantly improved overall survival and progression-free survival compared to placebo plus GC, with manageable safety [32].

The identification of biomarkers might lead to improved outcomes for patients with biliary tract cancer. The European Society for Medical Oncology Scale for Clinical Actionability of Molecular Targets (ESCAT) ranked genomic alterations in advanced cholangiocarcinoma based on therapeutic implications, and level I alterations included IDH1 mutations, FGFR2 fusions, microsatellite instability-high (MSI-H), and NTRK fusions [33]. The expression of PD-L1 in tumors has been proven to be associated with prolonged progression-free survival (hazard ratio, 0.23; 95% CI, 0.10–0.51; P < .001) [9]. Pembrolizumab is FDA-approved for advanced solid tumors, including BTC, that are mismatch-repair-deficient (dMMR)/ MSI-H or have a tumor mutational burden (TMB); TMB of 10 mutations per megabase or higher. Furthermore, exploratory subgroup analyses from the chemotherapy plus immunotherapy groups have indicated that mutations in CDKN2A and ARID1A might be associated with reduced progression-free survival compared to wild-type genes [34]. Therefore, further translational research is required to identify biomarkers that can help predict treatment response and prognosis.

This study is the first phase 2 study evaluating the efficacy and safety of sintilimab plus nab-paclitaxel in the second-line setting for advanced BTC, while also exploring predictive and prognostic biomarkers for treatment.

## Data Availability

Not applicable.

## Abbreviations

BTC: Biliary tract cancer
ECOG: Eastern Cooperative Oncology Group
PS: Performance status
ORR: Objective response rate
mOS: Median overall survival
PFS: Progression-free survival
NAB: Nanoparticle albumin-bound
ASC: Active symptom control
FOLFOX: Folinic acid, fluorouracil, and oxaliplatin
GC: Gemcitabine-cisplatin
ICI: Immune checkpoint inhibitor
RECIST: Response Evaluation Criteria in Solid Tumors guidelines
ULN: Upper limit of normal
PD-1: Programmed cell death-1
PD-L1: Programmed cell death-ligand 1
HIV: Human immunodeficiency virus
CRs: Complete responses
PRs: Partial responses
CTCAE: Common Terminology Criteria for Adverse Events
CT: Computed tomography
CEA: Carcinoembryonic antigen
CA199: Carbohydrate antigen 19 − 9
CA125: Carbohydrate antigen 125
HRQoL: Health-related quality of life
EORTC QLQ-C30: European Organization for Research and Treatment of Cancer Quality of Life Questionnaire Core 30
QLQ-BIL21: Cholangiocarcinoma and Gallbladder Cancer module
NGS: Next-generation sequencing
ChiCTR: Chinese Clinical Trial Registry (ChiCTR)
dMMR/MSI-H: Mismatch-repair-deficient/microsatellite instability-high
TMB: Tumor mutational burden
